# Supplementary material for: Melatonin delays ovarian aging in mice by slowing down the exhaustion of ovarian reserve
Source: Commun Biol. 2021 May 6;4:534. doi: 10.1038/s42003-021-02042-z (PMC8102596; doi:10.1038/s42003-021-02042-z)
Supplement: Supplementary file 1 — Supplementary Information [file 42003_2021_2042_MOESM1_ESM.docx]

**Supplementary Figures and Tables**

**Melatonin delays ovarian aging in mice by slowing down the exhaustion of ovarian reserve**

Chan Yang, Qinghua Liu, Yingjun Chen, Xiaodong Wang, Zaohong Ran*,* Fang Fang, Jiajun Xiong, Guoshi Liu, Xiang Li, Liguo Yang, Changjiu He

**
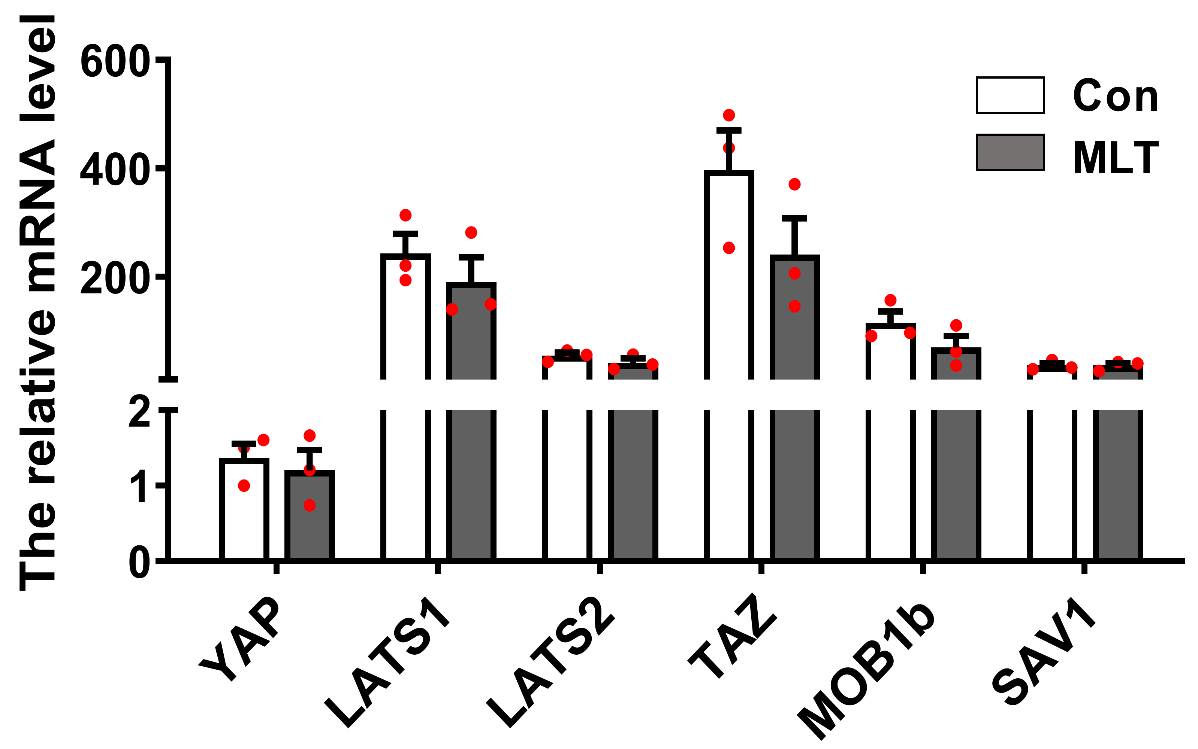
**

**Supplementary Fig. 1** **Effect of MLT injection on the expression of genes in Hippo pathway**. The data showed that MLT had no significant effect on the expression of genes in the Hippo pathway (values are mean ± S.E.M). Normalization was performed using the housekeeping gene *Actb*. n = 3 biologically independent mice. Statistical significance was determined using two-tailed unpaired Student’s t-test.


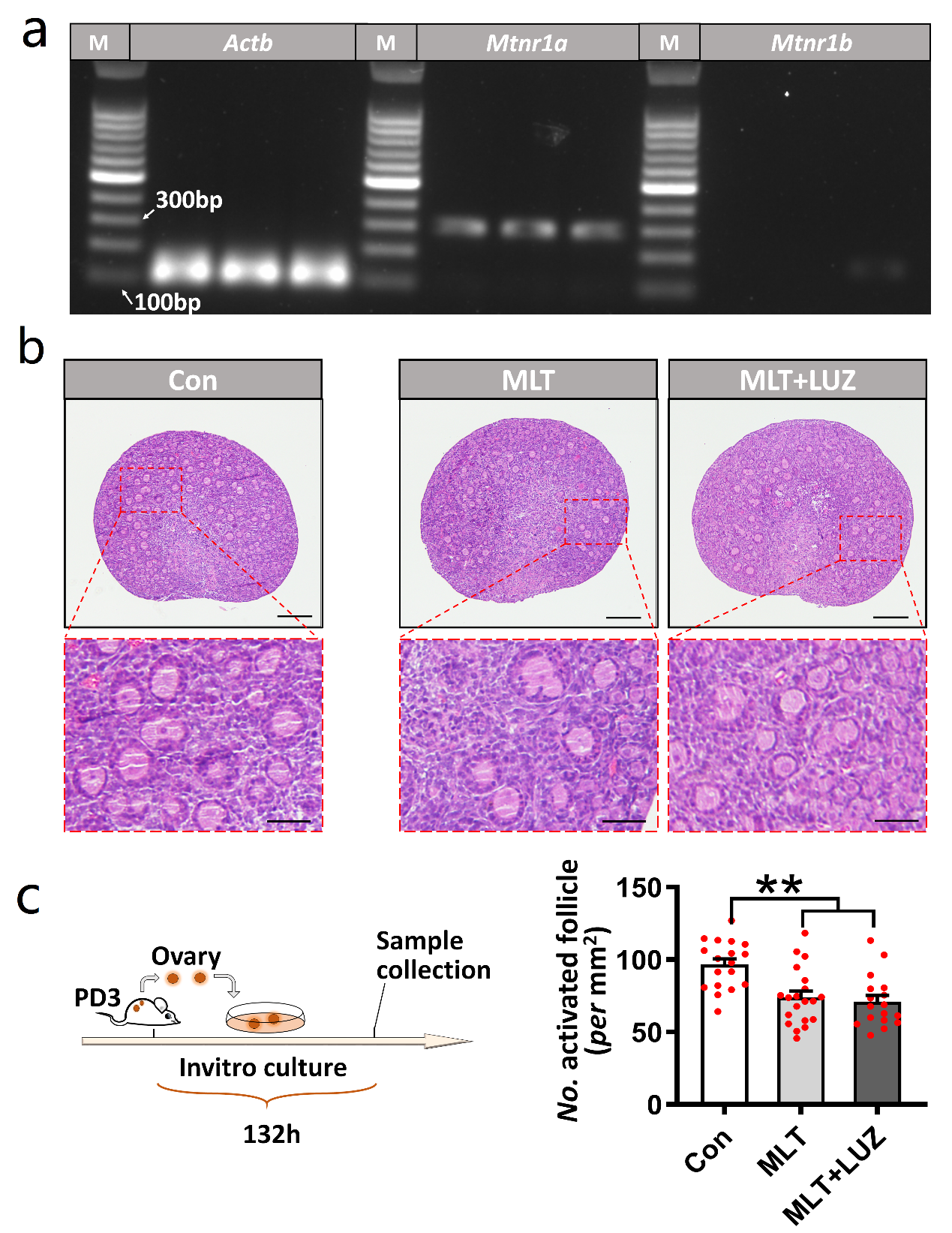


**Supplementary Fig. 2** **The inhibitory effect of melatonin on follicular activation is not mediated by its transmembrane receptors**. (a) RT-PCR revealed that MLT transmembrane receptor *Mtnr1a*, but not *Mtnr1b*, was expressed in neonatal mouse ovaries (PD6). Ovaries from three mice were used for assay. (b, c) Addition of Luzindole did not offset the inhibiting effect of MLT on follicle activation. n = 4 biologically independent ovaries. (b) Representative photographs of H&E staining in each group. The scale bars are 100µm in the original images and 40µm in the enlarged images. (c) Experimental design and statistical charts of activated follicles (values are mean ± S.E.M). The number of sections used for statistics: Con n=18, MLT n=20, MLT+LUZ n = 17. Statistical significance was determined using One-way ANOVA followed by Tukey post hoc test. Significant differences are denoted by **P <0.01.


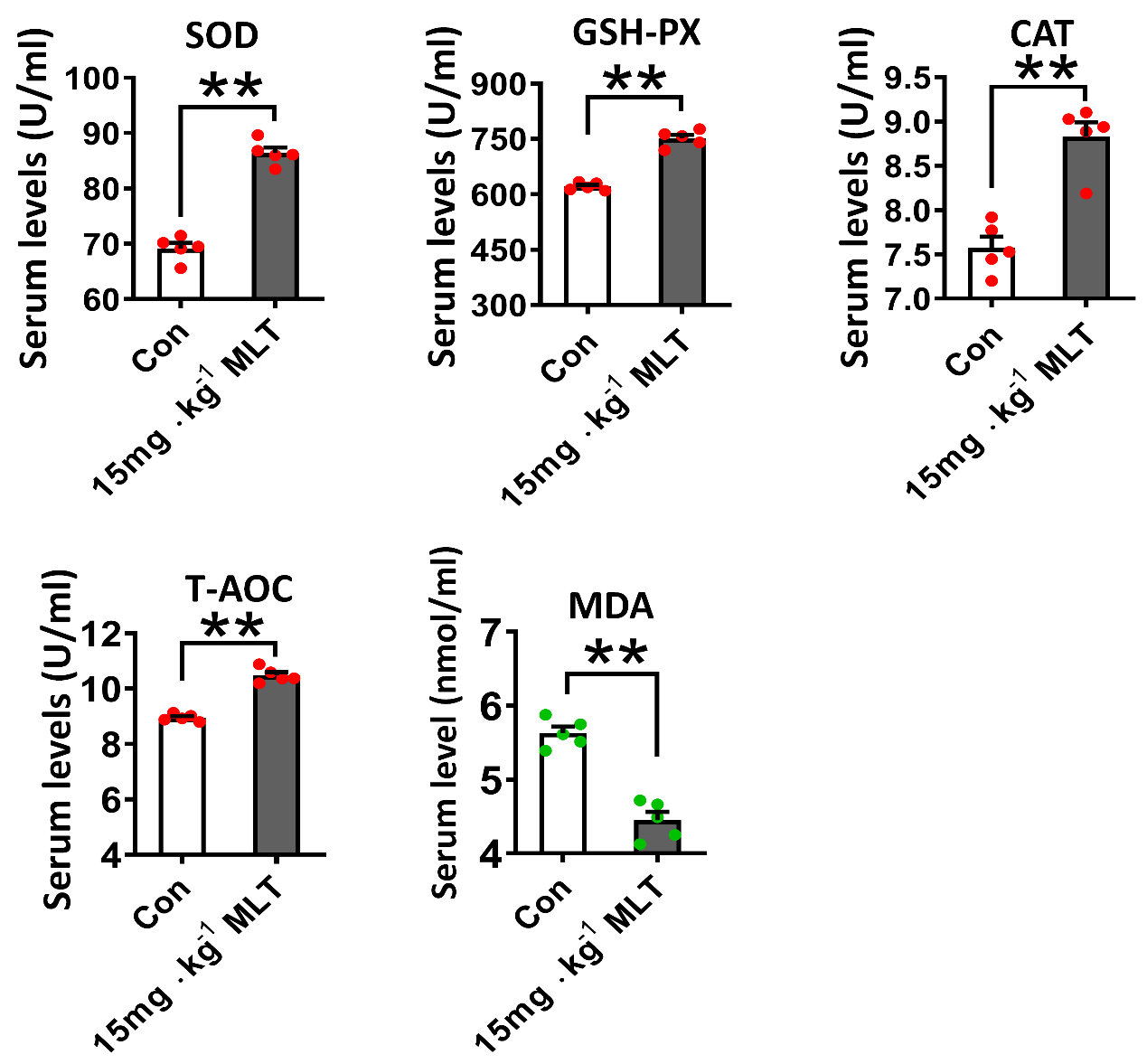


**Supplementary Fig. 3** **Effect of MLT injection on the serum levels of SOD, GSH-PX, CAT, T-AOC and MDA**. n = 5 biologically independent samples (values are mean ± S.E.M). Statistical significance was determined using two-tailed unpaired Student’s t-test. Significant differences are denoted by **P <0.01.

**
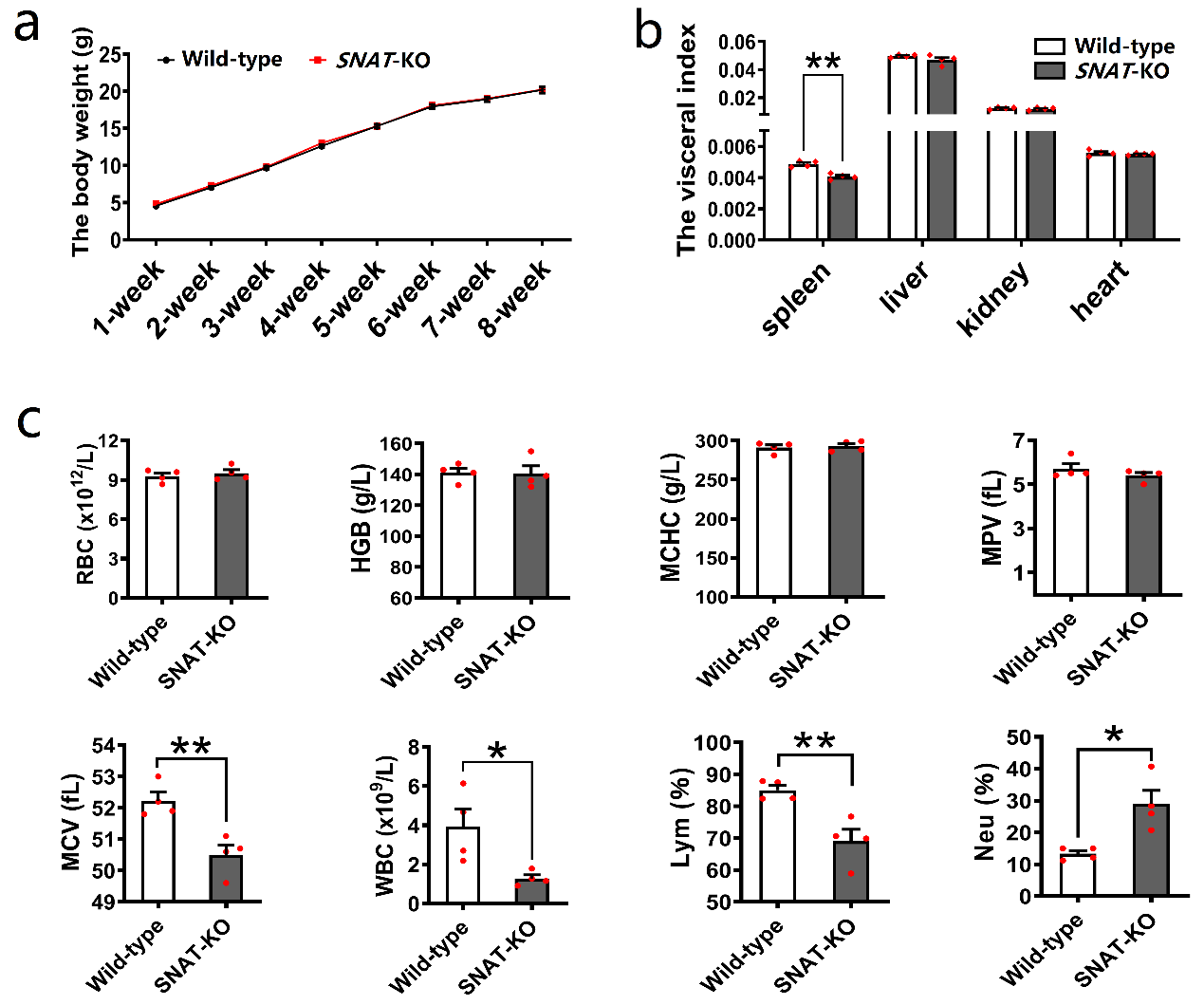
**

**Supplementary Fig. 4** **Effect of *SNAT* knockout on the body growth and heath condition of mice.** (a) Changes of weight gain after *SNAT* knockout (values are mean ± S.E.M). n = 17 (Wild-type), 15 (*SNAT*-KO) biologically independent mice, respectively. (b) Changes of viscera growth after *SNAT* knockout (values are mean ± S.E.M). The weight of spleen was decreased obviously. n = 4 biologically independent mice. (c) Changes of plasma biochemical indexes after *SNAT* knockout (values are mean ± S.E.M). Among them, mean corpuscular volume (MCV), white blood cell count (WBC), and lymphocyte proportion (Lym) were significantly decreased, while neutrophil percentage (Neu) was significantly increased. n = 4 biologically independent mice. Statistical significance was determined using two-tailed unpaired Student’s t-test. Significant differences are denoted by *P <0.05, and **P <0.01.

**Supplementary Fig. 5** Original Western blots in Fig. 3e

**
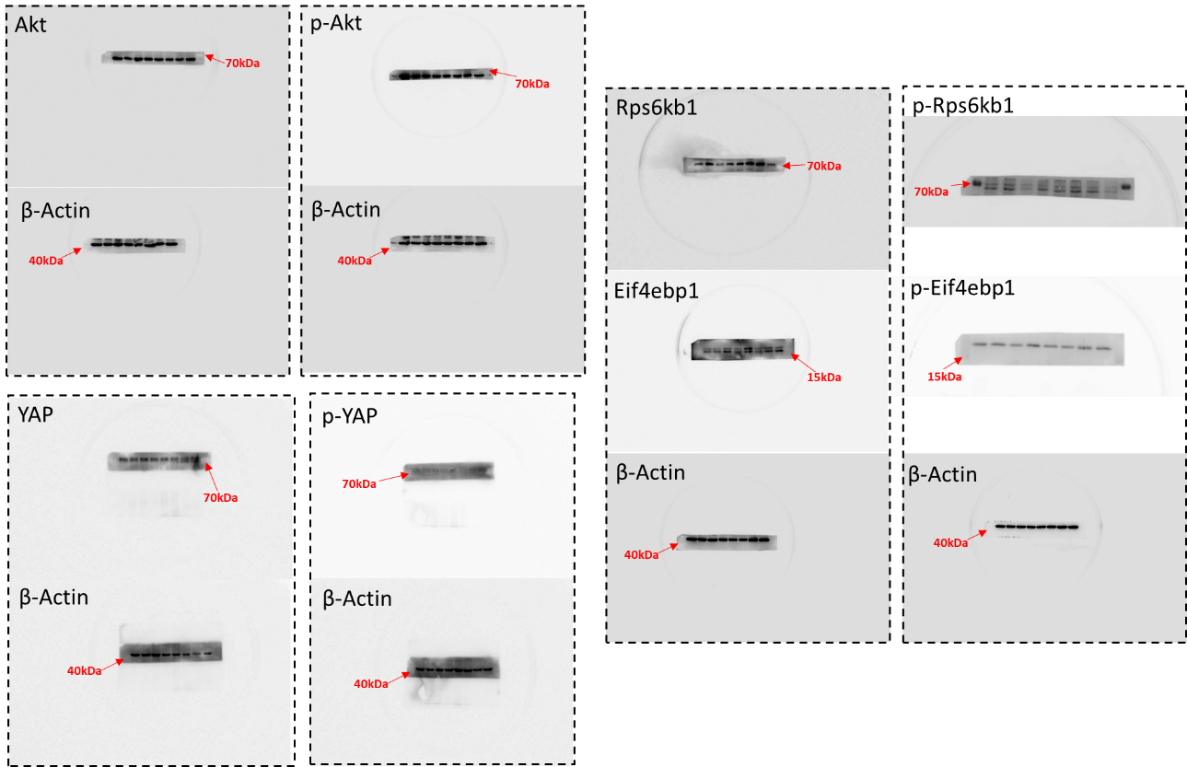
**

**Supplementary Fig. 6** Original Western blots in Fig. 4o


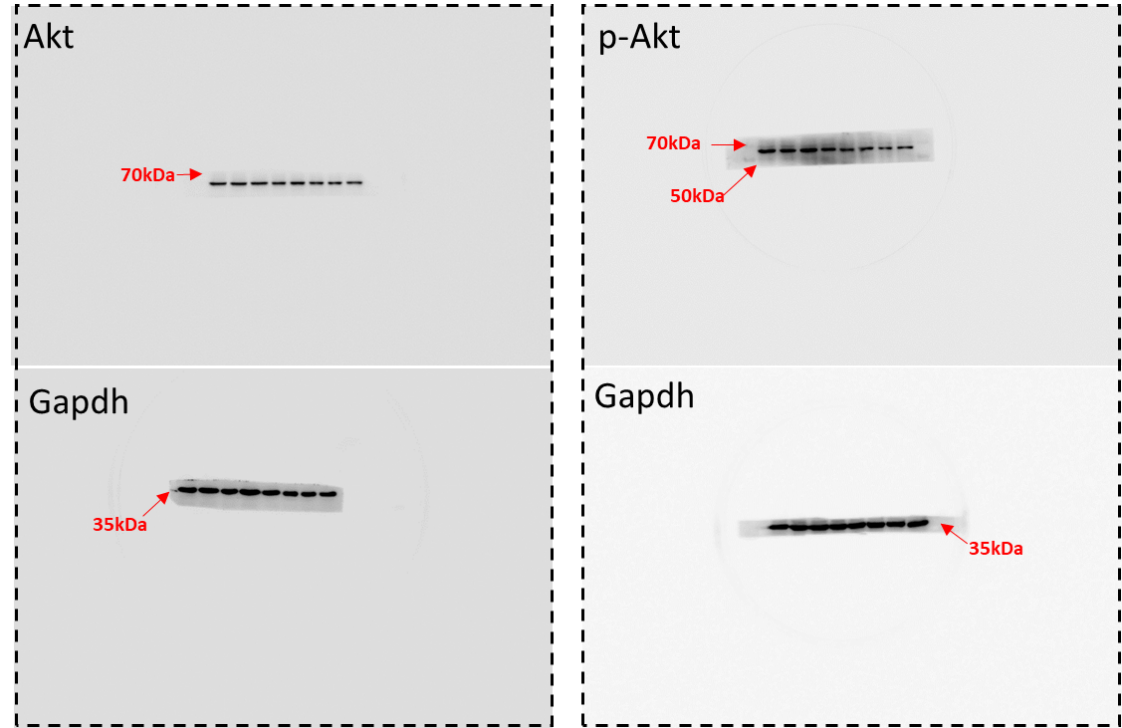


**Supplementary Fig. 7** Original Western blots in Fig. 5j

**
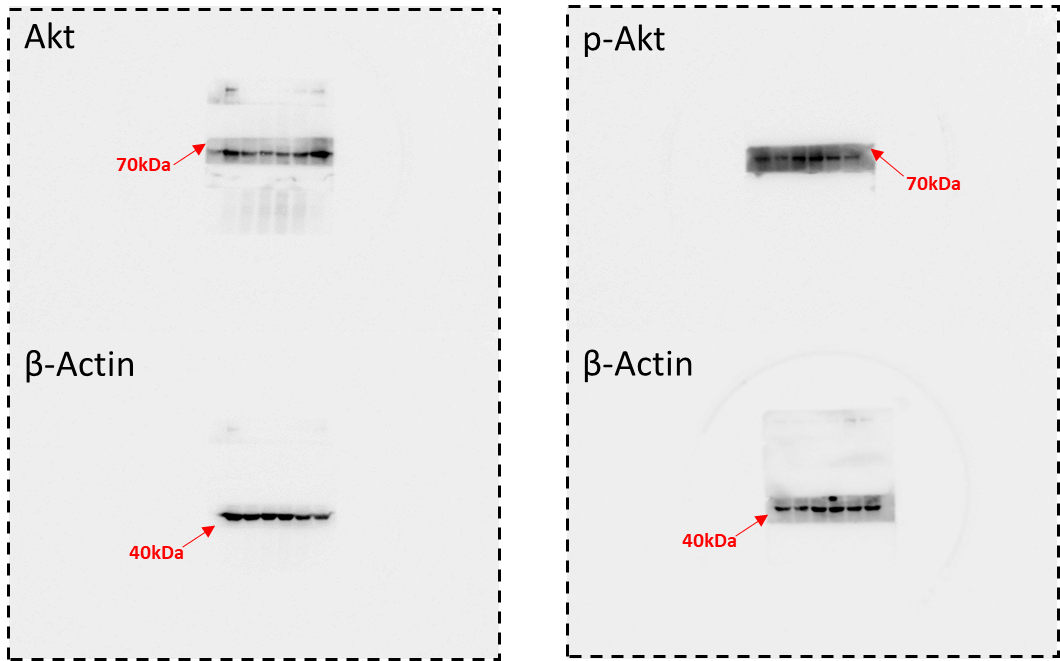
**

**Supplementary Table. 1** The primers used for quantitative PCR and RT-PCR

| **Genes** | **Primer sequence ( 5’-3’ )** | **Size ( bp )** |
| --- | --- | --- |
| *Actb* | Forward: CCAGCCTTCCTTCTTGGGTAT  Reverse: AGGTCTTTACGGATGTCAACG | 93 |
| *SNAT* | Forward: TGAACATCAACTCCCTGAAACCT  Reverse: TTCCCGCTCAATCTCAAACG | 152 |
| *Fshr* | Forward: GCAGATGTGTTCTCCAACCTACC  Reverse: GGAGAGACTGGATCTTGTGAAAGG | 172 |
| *Lhcgr* | Forward: CTGAGGAGATTTGGTTGCTGTA  Reverse: ATTTGGGTGGACTTTTTTGGGG | 234 |
| *PCNA* | Forward: ACCTGCAGAGCATGGACTCG  Reverse: GCAGCGGTATGTGTCGAAGC | 80 |
| *CAT* | Forward: CAGCGACCAGATGAAGCAGT  Reverse: CCTCAAAGTATCCAAAAGCACC | 236 |
| *SOD2* | Forward: CCAGACCTGCCTTACGACTATG  Reverse: CTCGGTGGCGTTGAGATTGT | 114 |
| *GSH-PX* | Forward: GGAGAATGGCAAGAATGAAGAG  Reverse: GGAAGGTAAAGAGCGGGTGA | 134 |
| *Figla* | Forward: CCGTTTCTACCACAGAGCAGG  Reverse: TTCTTCAAGCCACTCGCACA | 227 |
| *Nobox* | Forward: CCTTCAGTCACAGTTTCCGTATC  Reverse: GGGAGGTTCTGGCAAGCAAT | 226 |
| *Ddx4* | Forward: GTATTATTGTAGCACCAACTCG  Reverse: ACACCCTTGTACTATCTGTCG | 146 |
| *Mtnr1a* | Forward: AACCGTTACTGCTACATTTGC  Reverse: CTGAAGGACCAGGACCCATA | 279 |
| *Mtnr1b* | Forward: CTGAGACCGAGTGATTTGCG  Reverse: AAGAGCCCTTCTGGGACCTG | 146 |
| *YAP* | Forward: GCTGCCCGACTCCTTCTTCA  Reverse: TGGTACATCATCAGGGATCTCAAA | 247 |
| *LATS1* | Forward: AATTTGGCACACATCATAAAGCC  Reverse: ACGAGGGTCTTGGTAACTCATT | 233 |
| *LATS2* | Forward: CTTCCCTGGATGCCAAAGTCC  Reverse: GATAAGGTCCAAACTTCGGGG | 84 |
| *TAZ* | Forward: CAAATGGGTAGGAATTGGACG  Reverse: AGTAGGGTGGGCTGTTAGGG | 110 |
| *MOB1b* | Forward: ATGAGCTTCTTGTTTGGTAGTCG  Reverse: ATGACAGCCATCCGTAGGTTG | 137 |
| *SAV1* | Forward: CTGTCCCGCAAGAAAACCAAA  Reverse: AATGAAGGCATGAGATTCCGC | 107 |
| Primer1 | Forward: GATGAACGCCAGACTCTCCCTC  Reverse: AGCACAGTAAACAGAGCAGGCAG | 627 |
| Primer2 | Forward: AAGTAACCCAGACATCTGTCCC  Reverse: AGCACAGTAAACAGAGCAGGCAG | 669 |
