# Supplementary material for: Melatonin delays ovarian aging in mice by slowing down the exhaustion of ovarian reserve
Source: Commun Biol. 2021 May 6;4:534. doi: 10.1038/s42003-021-02042-z (PMC8102596; doi:10.1038/s42003-021-02042-z)
Supplement: Supplementary file 3 — Reporting Summary [file 42003_2021_2042_MOESM3_ESM.pdf]

nature research

Corresponding author(s): Changjiu He

Last updated by author(s): Feb 26, 2021

Reporting Summary

Nature Research wishes to improve the reproducibility of the work that we publish. This form provides structure for consistency and transparency in reporting. For further information on Nature Research policies, see our Editorial Policies and the Editorial Policy Checklist.

Statistics

For all statistical analyses, confirm that the following items are present in the figure legend, table legend, main text, or Methods section.

n/a	Confirmed	
☐	☒	The exact sample size (n) for each experimental group/condition, given as a discrete number and unit of measurement
☐	☒	A statement on whether measurements were taken from distinct samples or whether the same sample was measured repeatedly
☐	☒	The statistical test(s) used AND whether they are one- or two-sided<br>*Only common tests should be described solely by name; describe more complex techniques in the Methods section.*
☒	☐	A description of all covariates tested
☒	☐	A description of any assumptions or corrections, such as tests of normality and adjustment for multiple comparisons
☐	☒	A full description of the statistical parameters including central tendency (e.g. means) or other basic estimates (e.g. regression coefficient) AND variation (e.g. standard deviation) or associated estimates of uncertainty (e.g. confidence intervals)
☒	☐	For null hypothesis testing, the test statistic (e.g. F, t, r) with confidence intervals, effect sizes, degrees of freedom and P value noted<br>*Give P values as exact values whenever suitable.*
☒	☐	For Bayesian analysis, information on the choice of priors and Markov chain Monte Carlo settings
☒	☐	For hierarchical and complex designs, identification of the appropriate level for tests and full reporting of outcomes
☒	☐	Estimates of effect sizes (e.g. Cohen's d, Pearson's r), indicating how they were calculated

Our web collection on statistics for biologists contains articles on many of the points above.

Software and code

Policy information about availability of computer code

Data collection	No commercial, open source and custom code were used
Data analysis	Data were analyzed as described in the Methods section. Statistical analyses were performed using GraphPad Prism 7 (GraphPad, San Diego, CA, USA).

For manuscripts utilizing custom algorithms or software that are central to the research but not yet described in published literature, software must be made available to editors and reviewers. We strongly encourage code deposition in a community repository (e.g. GitHub). See the Nature Research guidelines for submitting code & software for further information.

Data

Policy information about availability of data

All manuscripts must include a data availability statement. This statement should provide the following information, where applicable:

- Accession codes, unique identifiers, or web links for publicly available datasets
- A list of figures that have associated raw data
- A description of any restrictions on data availability

The RNA-sequencing data was are openly available in Zenodo (DOI: 10.5281/zenodo.3365397). Source data underlying plots shown in figures are provided in Supplementary Data 1. All other data are available upon reasonable request.

Field-specific reporting

Please select the one below that is the best fit for your research. If you are not sure, read the appropriate sections before making your selection.

☒ Life sciences ☐ Behavioural & social sciences ☐ Ecological, evolutionary & environmental sciences

For a reference copy of the document with all sections, see nature.com/documents/nr-reporting-summary-flat.pdf

Life sciences study design

All studies must disclose on these points even when the disclosure is negative.

Sample size	Sample size was described in the figure legends.
Data exclusions	No data were excluded from the analyses.
Replication	The experimental techniques used in this study are all conventional techniques, and the experimental scheme and the reagents used are described in detail in EXPERIMENTAL PROCEDURES, which ensures the repeatability of this experiment on the technical level. The reproducibility was determined by using several biological replicates as indicated in the figure legends. Conclusions were not determined by a single detection technique, but by the combination of several experimental techniques from different perspectives. When performing histological analysis, we assigned three individuals who had not known grouping information to independently record, and obtained similar results.
Randomization	Except for the study of the effect of SNAT knockout on follicle activation and ovarian aging, the mice used in the other experiments were randomly divided into groups
Blinding	When analyzing sections, the statistics of morphological indicators such as activated follicles, growing follicles, and locked follicles are easily disturbed by subjective wishes. Therefore, we require observers to perform statistics without knowing the grouping information. The other experimental data can either be accurately counted by observers or obtained through software analysis, so the observers were not blinded to group allocation during analysis.

Reporting for specific materials, systems and methods

We require information from authors about some types of materials, experimental systems and methods used in many studies. Here, indicate whether each material, system or method listed is relevant to your study. If you are not sure if a list item applies to your research, read the appropriate section before selecting a response.

Materials & experimental systems

n/a	Involved in the study	
☐	☒	Antibodies
☒	☐	Eukaryotic cell lines
☒	☐	Palaeontology and archaeology
☐	☒	Animals and other organisms
☒	☐	Human research participants
☒	☐	Clinical data
☒	☐	Dual use research of concern

Methods

n/a	Involved in the study	
☒	☐	ChIP-seq
☒	☐	Flow cytometry
☒	☐	MRI-based neuroimaging

Antibodies

Antibodies used

rabbit anti-SNAT polyclonal antibody, Abcam, cat: ab3505, lot: GR263544-10
rabbit anti-Akt polyclonal antibody, Cell Signaling Technology, cat: 9272S, lot: 27
rabbit anti-p-Akt monoclonal antibody, Cell Signaling Technology, cat: 4060S, lot: 23
rabbit anti-Eif4ebp1 polyclonal antibody, Cell Signaling Technology, cat: 9452S, lot: 12
rabbit anti-YAP monoclonal antibody, Cell Signaling Technology, cat: 14074S, lot: 4
rabbit anti-p-YAP monoclonal antibody, Cell Signaling Technology, cat: 13008S, lot: 5
rabbit anti-p-Eif4ebp1 polyclonal antibody, Cell Signaling Technology, cat: 9451S, lot: 14
rabbit anti-p70s6k1 polyclonal antibody, Cell Signaling Technology, cat: 9202S, lot: 20
rabbit anti-p-p70s6k1 polyclonal antibody, Cell Signaling Technology, cat: 9205S, lot: 21
rabbit anti-Foxo3 monoclonal antibody, Cell Signaling Technology, cat: 2497S, lot: 8
mouse anti-β-actin monoclonal antibody, CWBiotech, cat: CW0096, lot: 01264/30339
mouse anti-GAPDH monoclonal antibody, CWBiotech, cat: CW0100, lot: 01225/50139
mouse anti-PCNA monoclonal antibody, Gene Tech, cat: GT2253
goat anti rabbit IgG, Servicebio Technology, cat: G1213
goat anti mouse IgG, Servicebio Technology, cat: G1214

	goat anti rabbit IgG, Servicebio Technology, cat: GB21303<br>goat Anti-mouse IgG (HRP), Biodragon-immunotech, cat: BF03001, lot: KIA7123684<br>goat Anti-rabbit IgG (HRP), Biodragon-immunotech cat: BF03008, lot: KIA8011698
Validation	Any validation and relevant citations of all antibodies can be found on the manufacturers' website according to the catalog number.

Animals and other organisms

Policy information about studies involving animals; ARRIVE guidelines recommended for reporting animal research

Laboratory animals	Female C57BL/6 mice (0-11 months old) ; Female KM mice (0-4-weeks old).
Wild animals	No wild animals were used in this study.
Field-collected samples	No field-collected samples were used in this study.
Ethics oversight	All experiments and animal handling were conducted in accordance with the institutional guidelines for animal experimentation after obtaining prior approval from the Institutional Animal Ethics Committee of Huazhong Agricultural University (HZAUMO-2017-049).

Note that full information on the approval of the study protocol must also be provided in the manuscript.
