# Supplementary material for: Melatonin delays ovarian aging in mice by slowing down the exhaustion of ovarian reserve
Source: Commun Biol. 2021 May 6;4:534. doi: 10.1038/s42003-021-02042-z (PMC8102596; doi:10.1038/s42003-021-02042-z)
Supplement: Supplementary file 5 — Description of Additional Supplementary Files [file 42003_2021_2042_MOESM5_ESM.pdf]

Description of Additional Supplementary Files

File name: Supplementary Data 1

Description: The source data underlying plots in all Figures.
